# Evaluation of the Social Effects of Enterprise Carbon Accounts Based on Variable Weight CFPR Fuzzy VIKOR

**DOI:** 10.3390/ijerph20043704

**Published:** 2023-02-19

**Authors:** Xiangyi Lin, Hongyun Luo, Yinghuan Lian, Yifei Jiang

**Affiliations:** 1 College of Business, Quzhou University, Quzhou 324000, China; 2 School of Economics and Management, Northeast Petroleum University, Daqing 163318, China; 3 School of Economics and Management, Beijing Forestry University, Beijing 100083, China

**Keywords:** carbon account, social effect, variable weight, CFPR, fuzzy VIKOR

## Abstract

The carbon account is a digital path for an enterprise to achieve low-carbon transformation and high-quality sustainable development under the ‘dual carbon’ strategy. The carbon account has a good social effect while generating economic benefits. An evaluation index system of the social effects of enterprise carbon accounts has been established, including the concepts of energy conservation and carbon reduction, contributions, technological innovation, and customer trust. In view of the difficulty of quantifying the evaluation indicators of the social effects of enterprise carbon accounts and the requirement of effect equalization, a variable-weight CFPR fuzzy VIKOR evaluation model was constructed. Compared with the traditional fuzzy VIKOR model, the variable-weight CFPR fuzzy VIKOR model can solve the problem of quantifying indicators and realize the balance between indicators. This method can better compare and analyze the social effects of each enterprise’s carbon accounts and provides a basis for overall carbon account construction and digging improvement space.

## 1. Introduction

On 22 September 2020, Chinese President Xi Jinping announced to the world at the general debate of the 75th United Nations General Assembly that ‘China’s carbon dioxide emissions will strive to reach the peak by 2030, and achieve carbon neutrality by 2060’ [1]. This indicates that the construction of China’s ‘double carbon’ strategy has begun on all fronts [2]. These detailed commitments demonstrate China’s determination to tackle climate change and play a key role in enhancing global sustainable development [3]. However, China is facing huge pressures in terms of energy conservation and emission reduction. In 2021, China’s carbon emissions were 10.523 billion tons, accounting for nearly 45% of the global total. In terms of total carbon emissions, industrial carbon emissions account for nearly 40% and are therefore the focus of China’s energy conservation and emission reduction strategies. As an important area for the implementation of China’s ‘double carbon’ strategy, Zhejiang Province paves the way for China’s energy conservation, carbon reduction, and high-quality economic development. Therefore, the practices of energy conservation and carbon reduction in Zhejiang Province are particularly concerning. There is no doubt that industrial energy conservation and carbon reduction are important measures for achieving the coordinated development of ‘double carbon’ and common prosperity in Zhejiang Province. As an important city in western Zhejiang Province, Quzhou is a city with a traditional heavy chemical industry. The heavy chemical industry in Quzhou accounts for nearly 70% of the city’s industry, and the carbon emissions from industrial sources account for more than 90% of the city’s total emissions. Industrial transformation and upgrading therefore face significant challenges [4].

In order to better conduct industrial energy conservation and carbon reduction and to accelerate the low-carbon transformation of industrial enterprises in order to achieve high-quality development, Quzhou has taken the lead in inventing enterprise carbon accounting systems in China. On 11 November 2022, at the 27th Conference of the Parties (COP27) to the United Nations Framework Convention on Climate Change, as a local practice case of climate change policies and actions, Quzhou’s carbon account finance appeared at the pavilion and was recognized by experts.

Through the construction and use of carbon accounts, enterprises have overcome three problems. First, intelligent data collection has solved the problems related to data collection and accounting linked to carbon emissions that are experienced in most regions due to difficulties in ensuring the authenticity, accuracy, and timeliness of carbon emission data through manual data collection. Second, by providing an accurate ‘carbon portrait’, it solved the issue that most enterprises’ carbon emissions accounting and carbon assessment methods are not sound and unified. Third, the ‘carbon incentive’ has improved the sense of gain of enterprises as social subjects in carbon-reduction and low-carbon strategies and enhanced enterprises’ participation and enthusiasm.

In recent years, Quzhou’s enterprise carbon accounts have generated better economic and social benefits in helping enterprises to save energy and reduce carbon. Economic benefits can be measured quantitatively through actual cost savings and profit growth. However, the social benefits of using enterprise carbon accounts are difficult to accurately quantify, and it is especially difficult to conduct a comparative analysis of social benefits among similar enterprises.

Therefore, the purpose of this paper was to construct an index system for the evaluation of the social effects of enterprise carbon accounts; we built a variable-weight CFPR fuzzy VIKOR model for assessing the social effects of enterprise carbon accounts and conduct a comparative analysis among similar enterprises.

The remainder of this paper is arranged into six sections. Section 2 presents the literature review. Section 3 details the concept of enterprise carbon accounts. Section 4 constructs an evaluation index system. Section 5 calculates the weight of the variable-weight fuzzy VIKOR model. Section 6 presents the case study, and, finally, Section 7, draws conclusions and details the limitations of the present model.

## 2. Literature Review

### 2.1. Digital Technology and Carbon Reduction

In the ‘digital’ era, the digital economy is known to have direct and indirect influences on carbon reduction [5]. Some experts suggest that increasing the digital economy index by 1% will cause CO_2_ emissions to decrease by 0.886% [6], which indicates that digital technology, digital innovation, and digital transformation are important for carbon neutrality targets. Digital technology can reduce carbon emissions by optimizing resource allocation, reducing energy consumption, and upgrading industrial structures [7]. Digital technology can promote carbon abatement through the ‘spillover effect’ [8]. Digital technology can enable many industries to realize digitalization transformations [9], monitor carbon emissions accurately, and reduce carbon emissions as far as possible. A system-generalized method of moments (SYS-GMM) technique can be used to assess the digital economy and its carbon-mitigation effects [6]. In summary, digital technology can first reduce unnecessary human activities, thus reducing carbon emissions. Second, digital technology can reshape production procedures and deeply mine potential carbon emissions, thus contributing to carbon emission reduction. In addition, digital technology can be used for monitoring, statistical analysis, and accounting of carbon emissions. All of these factors are the basis of enterprise carbon accounts.

### 2.2. Fuzzy MCDM for Evaluation

Many fuzzy methods are used for evaluation. AHP is a multicriteria decision-making technique, and as the generalization of the AHP, ANP can solve some problems that cannot be structured hierarchically because of their interactions with and dependence on each other [10]. Compared with AHP and ANP, consistent fuzzy preference relation (CFPR) has the advantages of sharply reducing pairwise comparison times, and it has high consistency throughout the calculation process [11]. These two methods require the subjective judgement of experts for decision making. Additionally, then, fuzzy AHP and ANP were presented based on fuzzy theory and AHP/ANP [12]. Some other methods, such as fuzzy TOPSIS, fuzzy VIKOR, the fuzzy Ordinal Priority Approach (fuzzy OPA), fuzzy cloud, and fuzzy DEMATEL, have been proposed for multi-criteria decision making [13,14,15]. Among these fuzzy MCDM methods, fuzzy cloud and fuzzy DEMATEL can be used to evaluate the absolute level of an alternative and analyze the potential reasons, while fuzzy TOPSIS, fuzzy VIKOR, and Fuzzy OPA are mainly applied to rank alternatives. The fuzzy TOPSIS and fuzzy VIKOR methods need a pairwise comparison, an ideal negative solution, and a positive solution. Fuzzy VIKOR can reflect group decision preferences by utility weight. Fuzzy OPA can be used to rank attributes and alternatives without a pairwise comparison or ideal solutions.

Some factors of the social effects of enterprise carbon accounts may exist in extremum intervals; when some factors reach extremum, the influencing effects are obvious. According to these reasons, this paper applies the variable-weight CFPR fuzzy VIKOR method to avoid the influencing effects detailed above.

## 3. Enterprise Carbon Accounts

### 3.1. Definition of Carbon Accounts

Enterprise carbon accounts refer to the comprehensive recording, scientific accounting, and fair evaluation of the carbon footprints of various enterprises. They have become a data governance tool, used to define the low-carbon contributions, carbon-reduction responsibilities, and carbon emissions ownership boundaries of social subjects. Quzhou uses carbon accounts as tools for digital governance to excavate the carbon trajectories of six key fields, including industry, agriculture (forestry), energy, construction, transportation, and residents’ lives. It has also established a carbon emission measurement system from production to consumption and developed a multi-dimensional carbon assessment to reflect industrial levels, regional contributions, and historical declines. This model can be applied to carbon finance, energy budget management, and ‘dual carbon’ technology, among other aspects. Quzhou has used the new generation of information technology to establish 2.396 million carbon accounts in six fields, including 100% of the above designated enterprises’ carbon accounts and 93% of individual carbon accounts.

### 3.2. Accounting Methods of Enterprise Carbon Accounts

The carbon emissions assessed in enterprise carbon accounts include direct and indirect carbon emissions. The direct carbon emissions are calculated using Formula (1), while the indirect carbon emissions are calculated using Formula (2):

*E*_direct_ = *E*_fossil fuel_ + *E*_industrial production_ (1)

*E*_fossil fuel_—carbon emissions from fossil fuel combustion.

*E*_industrial production_—carbon emissions from industrial production processes.

*E*_net purchased electricity_—carbon emissions from net purchased electricity.

*E*_indirect_ = *E*_net purchased electricity_ + *E*_net purchased heat_ (2)

*E*_net purchased heat_—carbon emissions generated by net purchased heat.

### 3.3. Evaluation Process of Enterprise Carbon Accounts

**Figure 1:**
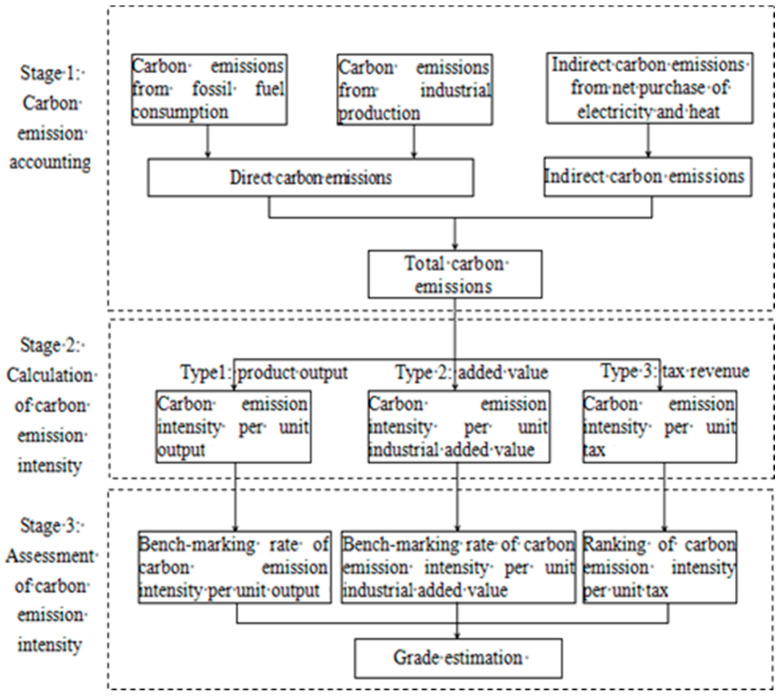
Process of enterprise carbon account evaluation.

The evaluation process of enterprise energy conservation and carbon reduction based on carbon accounts is divided into three stages, as shown in Figure 1.

The first stage mainly involves calculating the total carbon emissions of enterprises, including direct carbon emissions from fossil fuel consumption and industrial production, and indirect carbon emissions from net purchases of electricity and heat. The second stage mainly involves calculating carbon emission intensity, including carbon emission intensity per unit output, per unit industrial added value, and per unit tax. The third stage comprises evaluating the carbon emission intensity, including the benchmarking rate of carbon emission intensity per unit output and per unit industrial added value, and ranking carbon emission intensity per unit tax. According to these results, firms are given a carbon emissions grade, as shown in Table 1.

## 4. Evaluation Index System of the Social Effects of Enterprise Carbon Accounts

### 4.1. Construction of an Evaluation System for the Social Effects of Enterprise Carbon Accounts

The construction and application of carbon accounts by enterprises not only saves costs for enterprises, as can be measured effectively using quantitative methods, but also produces desirable social effects through energy conservation and carbon reduction. This has a significant impact on achieving the dual carbon target. However, as it is difficult to quantify the social effects generated in the application of enterprise carbon accounts; this paper constructs a social effect evaluation index system for enterprise carbon accounts from a qualitative perspective. Through discussion and exchange with some enterprises that use carbon accounts, as well as double carbon research experts and government officials, the evaluation index system of the social effects of enterprise carbon accounts was built around four main aspects. These mainly relate to the concepts of energy conservation and carbon reduction, the contributions of energy conservation and carbon reduction, the technological innovations related to energy conservation and carbon reduction, and customer respect and trust earned by energy conservation and carbon reduction, as shown in Table 2.

### 4.2. Interpretation of the Evaluation Index System for the Social Effects of Enterprise Carbon Accounts

(1) Concepts of energy conservation and carbon reduction. During the construction and application of enterprise carbon accounts, all employees of the enterprise are required to participate in this activity. The importance of carbon accounts in energy conservation and carbon reduction become well known, and everyone forms the habits of energy conservation and carbon reduction through the continuous diffusion of relevant knowledge to wider society by employees. Energy-conservation and carbon-reduction activities are organized spontaneously. Gradually, an enterprise- and society-wide atmosphere of energy conservation and carbon reduction is created.

(2) Contribution of energy conservation and carbon reduction. During the construction and application of enterprise carbon accounts, reasonable standards for the carbon emissions of various enterprises have been explored. This provided a basis for the formulation of national or industrial energy conservation and carbon reduction standards. The promotion and application of carbon accounts can greatly improve the energy consumption of society as a whole. Additionally, it can further reduce carbon emissions to promote the optimization of the ecological environment. The construction of carbon accounts also provides new ideas and specific practice paths for the transformation of the green, low-carbon, and high-quality development of enterprises.

(3) Technological innovation related to energy conservation and carbon reduction. The construction and application of enterprise carbon accounts must be upgraded through digital production processes. Energy consumption and carbon emission data should be obtained using intelligent methods. Enterprise production and management systems can be improved and optimized through digital technology innovation, and the continuous optimization of enterprise management methods can be promoted.

(4) Customer respect and trust earned by energy conservation and carbon reduction. The construction and application of enterprise carbon accounts greatly reduces the enterprise’s production processes and product carbon emissions. This also continuously reduces environmental pollution. The energy consumption levels and carbon emissions of products meet people’s everyday needs, improving enterprises’ reputations.

These four aspects reflect most of the social effects of enterprise carbon accounts, because enterprises that adopt carbon accounts can generate an atmosphere that is more conducive to energy conservation and carbon reduction. With carbon accounts, enterprise can make a significant contribution to China’s 2060 carbon neutrality target. At the same time, an improved atmosphere and technological innovation can earn customers’ trust, realizing energy conservation and carbon reduction by integrating production and consumption.

## 5. Calculation Process of the Variable-Weight Fuzzy VIKOR Model

In order to further compare and analyze the contributions and effects of using enterprise carbon accounts in Quzhou City, the contribution of each type of indicator is fully balanced, and extreme situations are avoided [16]. This paper uses a variable-weight fuzzy VIKOR model to evaluate the social effect of enterprise carbon accounts. Specifically, the secondary indicators use constant-weight coefficients based on fuzzy consistent preference relationships, and the primary indicators use variable-weight coefficients.

### 5.1. Defuzzification Steps of the Fuzzy VIKOR Method

VIKOR (Vlsekriterijumska Optimizacija I Kompromisno Resenie) is a multi-attribute decision-making method proposed by Opricovic and Tzeng in 1998. The VIKOR method obtains the compromising feasible solution that is closest to the ideal solution [17,18,19]. The VIKOR method comes from the aggregation function in *L_p_*-metric:

(3) $$L_{pi}={\left\{\sum_{j=1}^{n}{\left[w_{j}(f_{j}^{*}-f_{i}{}_{j})/(f_{j}^{*}-f_{j}^{-})\right]}^{p}\right\}}^{1/p}$$

where 1≤p≤+∞;i=1,2,…I. The VIKOR method not only overcomes the conflict of attributes and realizes mutual concessions between attributes, but it also maximizes the majority effect and minimizes individual objections. It reflects the subjective attitudes of evaluators by adjusting the value of the utility weight *v*. As a compromising multi-attribute group-decision-making method, the VIKOR method represents an effective evaluation tool for undertaking evaluation with preference information. Because people’s understanding, judgments, intuition, and preferences are sometimes fuzzy and difficult to measure, it is difficult to measure the value of evaluation indicators of the social effect of corporate carbon accounts. Meanwhile, fuzzy logic or fuzzy set theory can express and solve the expression of fuzzy index values in the evaluation of corporate carbon accounts’ social effects. Therefore, this paper proposes using a variable-weight fuzzy VIKOR method to evaluate the social effects of carbon accounts.

The fuzzy VIKOR method can give the evaluator a preference solution to the evaluation in the real organizational environment. The evaluation process is as follows [20,21].

Step 1: Determine feasible schemes, evaluation indicators, and experts. Suppose there are *m* enterprises to be evaluated, which are expressed by *A_i_* (*i* = 1, 2, …, *m*). *p* evaluation indicators are represented by *C_j_* (*j* = 1, 2, …, *p*), and *n* evaluation experts are represented by *E_k_* (*k* = 1, 2, …, *n*).

Step 2: Define the language variables and corresponding triangular fuzzy numbers. Language variables are used to determine the evaluation value of each scheme under different indicators, and are set for triangular fuzzy numbers $\tilde{A}=(a,b,c)$, where *a* < *b* < *c*. The membership function $f_{\tilde{A}}(x)$ corresponding to triangular fuzzy number $\tilde{A}$ is as follows:

(4) $$f_{\tilde{A}}(x)=\left\{ \begin{array}{l} 0,x<a\\ (x-a)/(b-a),a\leq x\leq b\\ (c-x)/(c-b),b\leq x\leq c\\ 0,x>c \end{array}\right.$$

Let $\tilde{A}=(a_{1},b_{1},c_{1})$ and $\tilde{B}=(a_{2},b_{2},c_{2})$. The calculation rules of triangular fuzzy numbers are as follows:

Addition rule: $\tilde{A}$ + $\tilde{B}$ = $\left(a_{1},b_{1},c_{1}\right)$ + $\left(a_{2},b_{2},c_{2}\right)$ = (*a*_1_ + *a*_2_, *b*_1_ + *b*_2_, *c*_1_ + *c*_2_)

Subtraction rule: $\tilde{A}$ − $\tilde{B}$ = $\left(a_{1},b_{1},c_{1}\right)$ − $\left(a_{2},b_{2},c_{2}\right)$ = (*a*_1_ − *a*_2_, *b*_1_ − *b*_2_, *c*_1_ − *c*_2_)

Multiplication rule: $\tilde{A}$ × $\tilde{B}$ = $\left(a_{1},b_{1},c_{1}\right)$ × $\left(a_{2},b_{2},c_{2}\right)$ = (*a*_1_*a*_2_,*b*_1_*b*_2_,*c*_1_*c*_2_)

$$k\tilde{A}=(ka_{1},kb_{1},kc_{1})$$

Division rule: $\tilde{A}$ ÷ $\tilde{B}$ = $\left(a_{1},b_{1},c_{1}\right)$ ÷ $\left(a_{2},b_{2},c_{2}\right)$ = (*a*_1_/*c*_2_,*b*_1_/*b*_2_,*c*_1_/*a*_2_)

$${\left(\tilde{A}\right)}^{-1}=(1/c_{1},1/b_{1},1/a_{1})$$

In this paper, the [0, 10] is used to determine the linguistic variables and corresponding triangular fuzzy numbers for the ranking of team members to be selected, as shown in Table 3.

Step 3: Calculate the fuzzy average evaluation value of the enterprises to be evaluated and construct a fuzzy evaluation matrix.

First, calculate the fuzzy average value of the *i*th enterprise to be evaluated under the *j*th index:

(5) $${\tilde{x}}_{ij}=\frac{1}{n}\left[\sum_{k=1}^{n}{\tilde{x}}_{ij}^{k}\right],i=1,2,\cdots ,m$$

Then, the fuzzy decision matrix is constructed:

(6) $$\tilde{D}={\left({\tilde{x}}_{i}{}_{j}\right)}_{m\times p}=\left[\begin{array}{cccc} {\tilde{x}}_{11} & {\tilde{x}}_{12} & \cdots & {\tilde{x}}_{1p}\\ {\tilde{x}}_{21} & {\tilde{x}}_{22} & \cdots & {\tilde{x}}_{2p}\\ \vdots & \vdots & \ddots & \vdots \\ {\tilde{x}}_{m1} & {\tilde{x}}_{m2} & \cdots & {\tilde{x}}_{mp} \end{array}\right],i=1,2,\ldots ,m;j=1,2,\ldots ,p$$

where ${\tilde{x}}_{i}{}_{j}$ is the fuzzy average value of the *i*th enterprise to be evaluated under the *j*th index expressed by a triangular fuzzy number.

Step 4: Defuzzification

The central value method is used to defuzzify the fuzzy evaluation matrix. The calculation formula of the central value method is as follows:

(7) $$D(\tilde{x})=\frac{2b+c+a_{}}{4}$$

### 5.2. Calculation of Constant Weight Value by CFPR

$W^{\left(0\right)}=(w_{1}^{\left(0\right)},w_{2}^{\left(0\right)},\cdots ,w_{m}^{\left(0\right)})(w_{j}^{\left(0\right)}>0(j=1,2,\cdots m),\sum_{j=1}^{m}w_{j}^{\left(0\right)}=1)$ represents the constant weight coefficient in the evaluation model of carbon accounts’ social effects, which is calculated based on the consistent fuzzy preference relationship (*CFPR*). The specific calculation process is as follows:

Proposition 1 [11,22,23]. Assumption scheme, set $X=\left\{x_{1},x_{2},\cdots ,x_{n}\right\}$ and its corresponding complementary multiplicative preference relationship $A=(a_{i}{}_{j})$, where $a_{ij}\in [1/9,9]$. The corresponding complementary fuzzy preference relation $P=(p_{ij})$, where $p_{ij}\in [0,1]$. The relationship between *P* and *A* is P=g(A), as shown below:

(8) $$p_{ij}=g(a_{ij})=\frac{1}{2}(1+\log_{9}(a_{ij}))$$

where *g* is the conversion function. The multiplicative preference relationship *A* can be connected with the fuzzy preference relationship *P* by the conversion function *g*. The corresponding conversion function can be selected according to the ratio scale used in the comparison of two alternatives. The conversion function used in Formula (8) is $\log_{9}(a_{ij})$, largely because $a_{ij}\in [1/9,9]$. If $a_{ij}\in [1/5,5]$; the conversion function *g* in Formula (8) should be $\log_{9}(a_{ij})$.

Proposition 2 [11,22,23]. For fuzzy preference relation $P=(p_{ij})$, then:

(9) $$p_{ij}+p_{jk}+p_{ki}=\frac{3}{2},\forall i<j<k$$

Proposition 3 [11,22,23]. For fuzzy preference relation $P=(p_{ij})$, then:

(10) $$p_{ij}+p_{jk}+p_{ki}=\frac{3}{2},\forall i<j<k$$

(11) $$p_{i(i+1)}+p_{\left(i+1)(i+2\right)}+\cdots +p_{(j-1)j}+p_{ji}=\frac{j-i+1}{2},\forall i<j$$

Proposition 3 is highly important and can be used to construct a consistent fuzzy preference relationship from *n* − 1 value sets $\left\{p_{12},p_{23},\cdots ,p_{(n-1)n}\right\}$ through additive transfer attributes after simple calculations. This not only greatly reduces the number of pairwise comparisons, but also always ensures the consistency of the data before and after using additive transfer attributes.

The value of the fuzzy preference relationship *p_ij_* calculated according to Formulas (9)–(11) may not fall in the interval of [0, 1]. However, it falls in the interval of [−*k*, 1 + *k*], where *k* > 0. At this time, we can use Formula (12) to convert the value of *p_ij_* into the interval of [0, 1], under the condition that the multiplicative complementarity and additive consistency of the data are completely guaranteed. By using the conversion function, the numerical conversion should be carried out according to the following steps [11]:

i. Calculate preference value set *B* of the decision matrix:

$$B=\left\{p_{ij},i<j\wedge p_{ij}\notin \left\{p_{12},p_{23},\cdots ,p_{(n-1)n}\right\}\right\}\;p_{ji}=\frac{j-i+1}{2}-p_{i(i+1)}-p_{\left(i+1)(i+2\right)}-\cdots -p_{(j-1)j}$$

ii. Calculate the *k* value:

$$k=|\min\left\{B\cup \left\{p_{12},p_{23},\cdots ,p_{(n-1)n}\right\}\right\}|$$

iii. Calculate *P*:

$$P=\left\{p_{12},p_{23},\cdots ,p_{(n-1)n}\right\}\cup B\cup \left\{1-p_{12},1-p_{23},\cdots ,1-p_{(n-1)n}\right\}\cup \neg B$$

iv. The conversion function of fuzzy preference relation $P^{\prime }=f(P)$ is as follows:
  (12) $$f:[-k,1+k]\to [0,1],f(x)=\frac{x+k}{1+2k},k>0$$
  (13) $$s_{i}=\frac{1}{n}\sum_{j=1}^{n}p_{ij}$$

The relative weight of each secondary indicator is calculated according to Formula (14):

(14) $$w_{i}=\frac{1}{n}\sum_{i=1}^{n}s_{i}$$

### 5.3. Calculation of the Variable Weight Value

The variable weight coefficient is adopted for the primary indicator of the social effect evaluation model of enterprise carbon accounts, and its calculation formula is as follows [16,24]:

(15) $$W_{j}(x_{1}^{},x_{2}^{},\cdots ,x_{m}^{})=w_{j}^{\left(0\right)}D{\left(\tilde{x}\right)}_{j}^{T-1}/\sum_{k=1}^{m}w_{k}^{\left(0\right)}D{\left(\tilde{x}\right)}_{k}^{T-1}$$

where *w_j_* is the variable weight coefficient of the *j*th state, $w_{j}^{\left(0\right)}$ is the constant weight coefficient of the *j*th primary indicator, $D{\left(\tilde{x}\right)}_{j}$ is the defuzzied value of the *j*th primary indicator, *m* is the number of primary indicators, and *T* is the variable weight coefficient (0 ≤ *T* ≤ 1).

When special attention is paid to a certain state quantity, *T* < 0.5. When the requirement for the balance degree of each state quantity is not high, *T* > 0.5. If the constant weight mode is selected, *T* = 1. The smaller the *T* value, the higher the degree of concern about the corresponding state quantity. The variable weight synthesis principle is an important modeling principle. It reflects the balance of the state of all elements in comprehensive decision making, because people will never adopt a scheme with strong necessity but poor feasibility, and vice versa. In other words, people always follow the principle of balance. Even if it is the least important factor, as long as the value is too small (or too large), the scheme will be abandoned.

### 5.4. Determination of the Ideal Solution and Ranking Alternations

Step 1: Determine the positive ideal solution $f_{j}{}^{*}$ and the negative ideal solution $f_{j}{}^{-}$

(16) $$f_{j}{}^{*}=\underset{i}{\max}x_{ij},i=1,2,\cdots ,m$$

(17) $$f_{j}{}^{-}=\underset{i}{\min}{\;x}_{ij},i=1,2,\cdots ,m$$

Step 2: Calculate *S_i_*, *R_i_*:

(18) $$S_{i}=\sum_{j=1}^{p}w_{j}(f_{j}^{*}-x_{i}{}_{j})/(f_{j}^{*}-f_{j}^{-})$$

(19) $$R_{i}=\underset{j}{\max}[w_{j}(f_{j}^{*}-x_{i}{}_{j})/(f_{j}^{*}-f_{j}^{-})]$$

*S_i_* represents the maximum group effect, and *R_i_* represents the individual regret value after the minimum.

Step 3: Calculate the *Q_i_* value of the enterprises to be evaluated, and rank the social effects of the carbon accounts of the enterprises to be evaluated according to the *Q_i_* value:

(20) $$Q_{i}=v(S_{i}-S_{}^{*})/(S^{-}-S^{*})+(1-v)(R_{i}-R^{*})/(R^{-}-R^{*})$$

where *Q_i_* represents the comprehensive score of social effects of the *i*th enterprise’s carbon account, *i* = 1, 2, …, *m*. $S^{*}=\underset{i}{Min}S_{i}$, $S^{-}=\underset{i}{Max}S_{i}$, $R^{*}=\underset{i}{Min}R_{i}$, $R^{-}=\underset{i}{Max}R_{i}$, and *v* is the maximum group utility weight. If *v* > 0.5, it indicates that most evaluators prefer the enterprise. If *v* = 0.5, it means that the evaluators have basically the same attitude. If *v* < 0.5, it means that most evaluators hold a negative attitude. If *v* = 0.5, it means that most evaluators hold a compromised attitude. The smaller the *Q_i_* value, the better the social effect of the enterprise’s carbon account, and vice versa.

## 6. Case Study

A system of enterprise carbon accounts was first implemented in Quzhou in Zhejiang Province. Therefore, this paper selects six enterprises above the designated size in Quzhou, Zhejiang Province, as the research object. The evaluation experts are composed of seven invited research experts, industrialists, and government officials in the dual carbon field. They evaluate the social effects of six enterprises that are in the process of applying carbon accounts.

The evaluation is divided into two stages. In the first stage, the experts use a 1–9 scale to score each primary indicator and secondary indicator according to the fuzzy consistent preference relationship and determine the indicator weight. In the second stage, the experts use the language variables shown in Table 3 to evaluate the social effects, which are generated in the application process of the six enterprises’ carbon accounts. Taking the four secondary indicators under the contribution of energy conservation and carbon reduction (*B*) as an example, the specific evaluation process is described.

### 6.1. Expert Language Evaluation Values of the Secondary Indicators (B)

According to the data provided by each enterprise and the on-site investigation, seven experts evaluated the four secondary indicators of B1, B2, B3, and B4 under the primary indicator of energy conservation and carbon reduction (B). This also follows the language variables in Table 3. See Table 4 for the evaluation results.

According to Formulas (5) and (6), the fuzzy mean values of the secondary indicators of energy conservation and carbon reduction (B) of the six evaluated enterprises are shown in Table 5.

### 6.2. Calculation of Evaluation Values and Constant Weight Values of Secondary Indicators of B

See Table 6 for the results after defuzzing the data in Table 5 according to Formula (7). According to Formulas (9)–(14), the constant weight values of the secondary indicators of energy conservation and carbon reduction (B) can be calculated, as shown in Table 6.

### 6.3. Calculation of the Evaluation Values and Variable Weight Values of the Primary Indicators

Based on the data in Table 6, we calculated the evaluation value (column 2 in Table 7) of the primary indicator (B) using the comprehensive weighting formula $\sum w_{j}v_{ij}$. Similarly, we can obtain the primary indicator values of the social effects of six enterprises’ carbon accounts, as shown in Table 7.

The constant weight value of the primary indicators of the social effects of the six enterprises’ carbon accounts is calculated according to Formulas (9)–(14). The variable weight value (*T* = 0.5) of the primary indicators of the social effects of the six enterprises’ carbon accounts is calculated according to Formula (15). See Table 8 for the calculation results.

### 6.4. Determination of the Ideal Solution and Ranking Alternations

Based on the data in Table 8, the positive and negative ideal solutions of the primary indicators for the social effect evaluation of six enterprises’ carbon accounts are calculated according to Formulas (16) and (17). See Table 9 for the calculation results.

The values of *S_i_*, *R_i_*_,_ and *Q_i_* are calculated according to Formulas (18)–(20). Generally, *v* is taken as 0.5, considering both the majority of the group effects and the minimum individual regret value. See Table 10 for the calculation results.

### 6.5. Comprehensive Analysis of the Evaluation Values

It can be seen from the data in Table 10 that the social effects of the six enterprises’ carbon accounts are ranked as follows: enterprise 1, enterprise 2, enterprise 5, enterprise 3, enterprise 6, enterprise 4. Despite being similar enterprises, the social effects of the six enterprises’ carbon accounts in the construction and application process are different. According to the *Q_i_* value, the social effects of the six enterprises’ carbon accounts are still quite different, and some enterprises have not taken advantage of their social effects. It is necessary to conduct in-depth analyses to identify the shortcomings and weaknesses according to the actual situation, so as to enhance the role of enterprise carbon accounts in energy conservation and carbon reduction at the social level.

Enterprise 4 is taken as an example for analysis. According to Table 8, among the variable weights of the primary indicators of the six enterprises’ carbon accounts’ social effect evaluation, the variable weight of Enterprise 4 and other enterprises’ primary-level indicators are compared. It was found that the variable weight of Enterprise 4’s primary indicator C is the highest, with a value of 0.261. Therefore, the primary indicator C plays an important role in the social effect evaluation of Enterprise 4’s carbon accounts. It can be seen from Table 7 that among the primary indicator values of the social effect evaluation of six enterprises’ carbon accounts, the value of indicator C is the lowest among the primary indicator values of Enterprise 4 compared with the other enterprises, with a value of 5.86. That is, the primary indicator C, with the highest weight for Enterprise 4, has the lowest carbon account social effect evaluation value among the six enterprises, which ultimately leads to Enterprise 4 having the minimum carbon account social effect.

Therefore, for Enterprise 4, we should attach great importance to the technical innovation of energy conservation and the carbon reduction of primary indicator C. We can begin with three aspects analyzed in this paper: promoting the digitalization of the production process, promoting intelligent energy data collection, and promoting the optimization of enterprise management methods. Enterprise 4 can collect data from the whole production process with the help of computers and can further expand this to the whole lifecycle of the product manufacturing process, which will bridge the gap between product design and product manufacturing and promote information integration. Enterprise 4 can use the energy big data exchange and sharing platforms of Zhejiang Province to improve intelligent energy-data-collection methods and promote the construction of application scenarios, such as ‘energy operation detection e-ledger’, ‘energy conservation and carbon reduction e-ledger’, and so on. By using these strategies in conjunction with the actual situation of Enterprise 4, the enterprise management methods can be gradually optimized. The technology and equipment used in the business’s processes can be used to achieve management objectives, such as energy conservation and carbon reduction. Additionally, new technologies can also be used to achieve these objectives.

## 7. Conclusions and Limitations

Our findings indicate that variable weight can reflect which indicator’s influence is important; that is, the greater the variable weight value, the greater the influence of the factors. In the final ranking of alternatives, the variable-weight value plays the most important role. The variable-weight CFPR fuzzy VIKOR is fit for evaluating the social effects of enterprise carbon accounts.

The limitation of this paper is that the index system is constructed from the perspective of qualitative measurement. Enterprise carbon accounts are adopted to decrease carbon emissions through the use of digital technology. The social effects of enterprise carbon accounts can mainly be reflected by qualitative indexes. If enterprise carbon accounts are optimized and popularized in China, we can identify quantitative indicators to replace some of the constructed index indicators in the future.

## Figures and Tables

**Table 1:** Classification of carbon emissions intensity per unit output.

FLAG COLOR	Strength Grade	Benchmarking Rate	Grade Description
Dark green	A	0, 50%	Excellent
Light green	B	50%, 100%	Good
Yellow	C	100%, 120%	Secondary
Red	D	120%, +∞	Poor

**Table 2:** Evaluation index system of the social effects of enterprise carbon accounts.

Order Number	Primary Indicators	Secondary Indicators
A	Concepts of energy conservation and carbon reduction	A1 Publicize awareness of energy conservation and carbon reduction A2 Spread knowledge of energy conservation and carbon reduction A3 Create an atmosphere of energy conservation and carbon reduction
B	Contribution of energy conservation and carbon reduction	B1 Promote the formulation of national or industrial energy conservation and carbon reduction standards B2 Promoting social energy consumption and saving B3 Reduce carbon emissions and promote optimization of the ecological environment B4 Promote the transformation of green, low-carbon, and high-quality development
C	Technological innovations related to energy conservation and carbon reduction	C1 Promote the digitalization of the production process C2 Promote intelligent energy data collection C3 Promote the optimization of enterprise management methods
D	Customer respect and trust earned by energy conservation and carbon reduction	D1 Improve enterprise reputation D2 Ensure that the carbon emission standards of products meet consumer demands

**Table 3:** Language variables and corresponding triangular fuzzy numbers for the ranking of the enterprises to be evaluated.

Language Variables	Triangular Fuzzy Numbers
Very weak (VW)	(0, 0, 2.5)
Weak (W)	(0, 2.5, 5)
Medium (M)	(2.5, 5, 7.5)
good (G)	(5, 7.5, 10)
Very good (VG)	(7.5, 10, 10)

**Table 4:** Expert language evaluation values of the secondary indicators of energy conservation and carbon reduction (B) of the six evaluated enterprises.

		B1	B2	B3	B4
Enterprise 1	E1 E2 E3 E4 E5 E6 E7	G G M VG VG W VG	G VG VG G M M G	M M G W W G VG	M G G VG M M W
Enterprise 2	E1 E2 E3 E4 E5 E6 E7	M VG G M M G W	M G W G VG G G	G G W M W M M	VG M W W G G M
Enterprise 3	E1 E2 E3 E4 E5 E6 E7	G G M VG M W W	W G M VG G G VG	G G G M W G G	VG M G VG G VG M
Enterprise 4	E1 E2 E3 E4 E5 E6 E7	G G M G G G W	M G W VG M M G	G VG W M W G M	G G VW W G G M
Enterprise 5	E1 E2 E3 E4 E5 E6 E7	M VG G G M G G	M VG W G VG VG G	VG G VG M W G M	VG M VW W G M M
Enterprise 6	E1 E2 E3 E4 E5 E6 E7	M G G VG M G W	M G W VG G VG G	VG G W W W M M	VG M M G G G M

**Table 5:** Fuzzy mean values of the secondary indicators of energy conservation and carbon reduction (B) of the six evaluated enterprises.

	B1			B2			B3			B4		
Enterprise 1	5.0	7.5	8.9	5.0	7.5	9.3	3.2	5.7	7.9	3.6	6.1	8.2
Enterprise 2	3.6	6.1	8.2	4.3	6.8	8.9	2.5	5.0	7.5	3.2	5.7	7.9
Enterprise 3	3.2	5.7	7.9	4.6	7.1	8.9	3.9	6.4	8.9	5.4	7.9	9.3
Enterprise 4	3.9	6.4	8.9	3.6	6.1	8.2	3.2	5.7	7.9	3.2	5.4	7.9
Enterprise 5	4.6	7.1	9.3	5.0	7.5	8.9	4.3	6.8	8.6	2.9	5.0	7.1
Enterprise 6	3.9	6.4	8.6	4.6	7.1	8.9	2.5	5.0	7.1	4.3	6.8	8.9

**Table 6:** Evaluation values of the secondary indicators of energy conservation and carbon reduction (B) of the six evaluated enterprises after defuzzification.

	B1	B2	B3	B4
Constant weight values	0.201	0.239	0.212	0.248
Enterprise 1	7.23	7.33	5.63	6.00
Enterprise 2	6.00	6.70	5.00	5.63
Enterprise 3	5.63	6.93	6.40	7.63
Enterprise 4	6.40	6.00	5.63	5.48
Enterprise 5	7.04	7.23	6.61	5.00
Enterprise 6	6.33	6.96	4.91	6.70

**Table 7:** Primary indicator values of the social effects of six enterprises’ carbon accounts.

	A	B	C	D
Enterprise 1	6.12	5.89	6.89	6.87
Enterprise 2	5.64	6.27	6.22	6.37
Enterprise 3	6.12	6.04	6.17	6.04
Enterprise 4	5.87	6.27	5.86	5.96
Enterprise 5	6.01	6.78	6.75	5.98
Enterprise 6	6.53	5.64	6.64	6.27

**Table 8:** Variable weights of the primary indicators for the social effect evaluation of six enterprises’ carbon accounts.

	A	B	C	D
Constant weight	0.213	0.274	0.258	0.255
Enterprise 1	0.218	0.286	0.249	0.247
Enterprise 2	0.222	0.271	0.256	0.250
Enterprise 3	0.212	0.275	0.256	0.256
Enterprise 4	0.215	0.268	0.261	0.256
Enterprise 5	0.220	0.266	0.251	0.264
Enterprise 6	0.208	0.288	0.250	0.254

**Table 9:** Positive and negative ideal solutions of the primary indicators for the social effect evaluation of six enterprises’ carbon accounts.

	$\mathrm{Positive}\;\mathrm{Ideal}\;\mathrm{Solution}\;f_{j}{}^{*}$	$\mathrm{Negative}\;\mathrm{Ideal}\;\mathrm{Solution}\;f_{j}{}^{-}$
*A*	6.53	5.64
*B*	6.78	5.64
*C*	6.89	5.86
*D*	6.87	5.96

**Table 10:** Values of *S_i_*, *R_i_*, and *Q_i_*.

	*S_i_*	*R_i_*	*Q_i_*	Sort
Enterprise 1	0.324	0.223	0.009	1
Enterprise 2	0.648	0.222	0.343	2
Enterprise 3	0.689	0.234	0.473	4
Enterprise 4	0.796	0.261	0.795	6
Enterprise 5	0.420	0.258	0.372	3
Enterprise 6	0.516	0.288	0.704	5

## Data Availability

The data that support the findings of this study are available upon request to the corresponding author.
